# Influence of WeChat on sleep quality among undergraduates in Chongqing, China: a cross-sectional study

**DOI:** 10.1186/s40064-016-3730-z

**Published:** 2016-12-01

**Authors:** Xianglong Xu, Qianyi Lin, Yan Zhang, Runzhi Zhu, Manoj Sharma, Yong Zhao

**Affiliations:** 1 School of Public Health and Management, Chongqing Medical University, No. 1 Yixueyuan Road, Yuzhong District, Chongqing, 400016 China; 2 Research Center for Medicine and Social Development, Chongqing Medical University, Chongqing, 400016 China; 3 Collaborative Innovation Center of Social Risks Governance in Health, Chongqing Medical University, Chongqing, 400016 China; 4 School of the Second Clinical, Chongqing Medical University, No. 1 Yixueyuan Road, Yuzhong District, Chongqing, 400016 China; 5 Department of Behavioral and Environmental Health, Jackson State University, Jackson, MS 39213 USA

**Keywords:** WeChat, Sleep quality, Undergraduates, Social media, China

## Abstract

**Background:**

Previous studies showed that social media is associated with sleep quality. WeChat (a native social media in China) is very popular in China, especially among the youth. In the second quarter of 2016, Tencent’s WeChat had 806 million monthly active users. The study sought to identify the influence of WeChat on the sleep quality among undergraduate students.

**Methods:**

A cross-sectional survey adopted a multi-stage stratified sampling survey to investigate undergraduates in Chongqing, China. Data were collected on 1979 eligible adults, aged 20.27 (SD: 1.26) years old, using Pittsburgh Sleep Quality Index (PSQI) to measure sleep quality.

**Results:**

Respondents aged 20.27 ± 1.26 years included 535 (27.0%) males, and 1311 (66.3%) reported as having poor sleep quality. Of the 1979 participants, 1320 (66.70%) were WeChat users. In multivariable analyses, gender, grade, nationality, living costs, the student leader, the only child, type of university, WeChat usage was associated with domains of PSQI among undergraduates (*p* < 0.05 for all). Compared with non-users, WeChat users had a lower score of subjective quality of sleep, sleep latency, use of sleeping medication, daytime dysfunction, and global PSQI score (*p* < 0.05 for all).

**Conclusions:**

WeChat users may have better sleep quality than non-WeChat users among undergraduates. To determine causal relationships, further longitudinal studies will be required to test for the association between WeChat users and sleep quality. This study may also provide some implications for health promotion on sleep quality of undergraduate students.

## Background

In recent years, China has witnessed an unprecedented boom in the use of social media. WeChat (Wikipedia 2011), first launched in January 2011, is a mobile communication tool developed by Tencent in China. As an instant message application, WeChat mainly focuses on the communication functions like sending text, voice, video and photo messages, group chat, scanning quick response code to add friends, and finding new friends nearby with advancement such as supporting moments (a built-in platform for sharing photos, texts, and links to friends), games, mobile payment, subscriptions of official accounts (providing service or contents by third-party individuals or organizations), and other assistant functions (Chen 2015). WeChat would include some new features compared to Whatsapp, a more popular social media in western countries. It has integrated video calling option which is not the case with Whatsapp. Differently from WeChat, it is possible for Skype users to make phone calls to land lines and cellphones around the world, at rates that are fixed and generally low. Facebook is now, by a very wide margin, which allows its users to create their own Facebook page, add friends and share personal updates and photos. WeChat is a type of social media that has gained considerable popularity among the young population in China. In the second quarter of 2016, Tencent’s WeChat had 806 million monthly active users (The Statistics Portal 2016). There was a significant increase in frequency and long duration of WeChat use and 25% users checking WeChat more than 30 times per day, according to the user and platform data shared by Tencent in January 2015 (Tencent 2015).

Sleep problems and related daytime impairment are risk factors for numerous problems among college students including poor physical and mental health (Lund et al. 2010; Taylor et al. 2011), decrements in academic performance (Gaultney 2010), difficulty concentrating, and poorer lifestyle factors. Poor sleep quality among college students is also predictive of problem drinking (Kenney et al. 2012), and suicidal thoughts (Nadorff et al. 2011). In a 2012 American national survey of college students, 26.4% reported sleep difficulties during the previous 12 months as being “traumatic or very difficult to handle”, and 57.1% of the sample reported having enough sleep to feel rested on fewer than 4 days a week (ACHA 2012). Lack of sleep has been one of the most important public health problems in China and been neglected for a long time. Undergraduate students suffer from sleep problems around the globe (Lund et al. 2010), as well as in China more specifically (Suen et al. 2008). Most college students are sleep deprived, as 70.6% of students report obtaining <8 h of sleep (Lund et al. 2010). A similar study among Chinese college students reported an average duration of sleep during weekdays to be 6.9 h (Tsui and Wing 2009). The prevalence of poor sleep quality was 54.8% among Chinese college student population in 2015 (Huang et al. 2015). In a Chinese study, 19% of the medical students were found to have poor sleep quality as assessed by the Pittsburgh Sleep Quality Index (PSQI), with differences seen between years of study but not between genders (Feng et al. 2005).

Sleep issues may be associated with other student risk behaviors related to voluntary lifestyle choices including drinking patterns (Wechsler et al. 2000), sexual activities (Clemmens et al. 2004) and other lifestyle choices such as physical activity, and increasing use of technology, increased work and social demands (Chokroverty 2009; Trockel et al. 2000). Poor sleep quality was found to be associated with less life satisfaction, habit of delayed sleep and no breakfast, negative life events, perceived stress level and symptoms of depression (Lemma et al. 2012; Liu et al. 1995). Today’s university students experience great psychological pressure due to the changing career market and increased competition for jobs (Biggeri et al. 2001). However, the timing and expression of sleep and wakefulness are highly influenced by environmental factors (Buysse et al. 2003). Although there might be significant variations from campus to campus, college culture is characterized by social arrangements and behavior practices that negatively affect students’ sleep, including communal living, frequent parties and drinking, poor time management, and high levels of stress and work (Buboltz et al. 2002; Jensen 2003; Wechsler et al. 2000). Lifestyle, social and academic schedules, and insufficient sleep education could all contribute to the aetiology of chronic sleep insufficiency and poor sleep in university students (Brown et al. 2002, 2006; Buboltz et al. 2001).

Previous studies showed that social media is associated with sleep quality. A study in Flanders, Belgium showed that there was no safe dose and no safe time for using the mobile phone for text messaging or for calling after lights out (Van den Bulck 2007). A previous study has shown that the prevalence of Facebook dependence is associated with poor sleep quality (Wolniczak et al. 2013). Higher social media use volume and frequency had significantly greater odds of having sleep disturbance among young adults (Levenson et al. 2016). Smartphone overuse may lead to depression and/or anxiety, which in turn leads to sleep problems (Demi̇rci̇ et al. 2015). Excessive use of social media might result in misuse, dependence, and addiction (Griffiths 2012). A possible mechanism for the association of social media use and sleep quality is that the continuous light stimulation of an electronic screen could be sleep quality (Liu and Zhao 2014) as it altering the circadian rhythm, leading to insomnia and excessive sleepiness (Barion and Zee 2007). Moreover, a decrease in sleep duration leads to an increase in daytime sleepiness, influencing learning efficiency and academic performance, especially for undergraduates (Gomes et al. 2011).

However, no study has yet investigated the association between sleep quality and WeChat use among Chinese undergraduates. To our knowledge, the data regarding sleep patterns and habits in the Chinese undergraduate are limited. The effects of WeChat on the sleep quality of undergraduate students have not been studied thoroughly. Clarifying the effects of WeChat on the sleep quality of undergraduate students is necessary for the further exploration of the association between social media and sleeping problems particularly in China. The purpose of this study was to identify the relationship between WeChat use and sleep quality of undergraduate students, and increase the awareness of undergraduate about the importance of adopting healthy sleeping habits by addressing three research questions as follows: (1) What are the factors that affect WeChat usage among college students in China; (2) the effect of WeChat on sleep quality, (3) whether using WeChat has serious consequence on quality of sleeping.

## Methods

### Study design

The study design and methods, including the population and sample and the sampling framework, as well as the survey administration, pilot study and questionnaire development process have been reported previously (Xu et al. 2015). A cross-sectional study was conducted on undergraduates in Chongqing University Town. Chongqing University Town is located in Shapingba district, about 15 km from the downtown area of Chongqing. According to literature (Cheng et al. 2012), the prevalence of poor sleep quality was 54.7% of the total student population. We set P = 0.547. ($$ P = 0.547; $$ $$ Q = 1 - P = 1 - 0.547 = 0.453 $$ margin of error $$ d = 0.1,\,P = 0.1 \times 0.547 = 0.0547,\,Z_{\alpha } = 1.96; $$ the sampling size is $$ N = \frac{{Z_{\alpha }^{2} \times pq}}{{d^{2} }} = \frac{{1.96^{2} \times 0.547 \times 0.453}}{{0.0547^{2} }} = 318 $$; sampling size = 318). The respondents were all students attending the selected classes. Respondents included 2100 volunteer undergraduate students, who were selected through probability proportionate sampling. Among the 2100 students, the response rate was 2088 (99.43%). Nine responses were deleted because missing data, and thus the final sample comprised 1979 (94.24%) respondents.

### Ethical approval

The study was conducted in accordance with the Declaration of Helsinki, and the protocol was approved by the Ethics Committee of Chongqing Medical University (2013036). The protocol and procedures were explained verbally to each subject prior to obtaining written consent to participate. A pilot study was conducted in June 2014 to test the feasibility of the proposed study.

### Questionnaire

The present study used the PSQI to assess sleep quality, which is widely accepted as both valid and reliable (Trockel et al. 2000) and has been previously used to assess undergraduate students in Taiwan (Kang and Chen 2009). The Cronbach alphas of global PSQI in this study was found to be 0.643, the reliability is good. The collected information included:

1. Demographic information from each subject, such as type of university (Comprehensive University/University of Foreign Languages/Medical University/Normal University/Technological University), student leader, age, gender, grade, living expenses, and race; according to the Ministry of Education on the division of subjects and subjects’ proportion in a university, universities can be divided into 13 different kinds (Wu 2002). University student leaders organize and coordinate common students to realize the goal of university education within the scope of duties and authority management (Shi 2008).
2. The questionnaire provided a screening instrument for using WeChat: 1. Do you use WeChat more than once in the recent week? (yes/no)
3. PSQI; Component 1: subjective quality of sleep; Component 2: sleep latency; Component 3: sleep duration; Component 4: habitual sleep efficiency; Component 5: sleep disorders; Component 6: use of sleeping medication; Component 7: daytime dysfunction. The PSQI includes 19 items, and yields a score from 0 (good quality) to 21 (poor quality). Sleep onset latency and sleep efficiency, defined as the actual sleep time divided by the time in bed, are also obtained with the PSQI.
4. How social media affects sleep quality, including sleep onset latency, multiple arousals during sleep, waking up in the morning, memory, mood, fatigue at work, and feeling upset at daytime.

### Data analysis

The characteristics of the participants were summarized using either means and standard deviations or frequencies and percentages. Chi square tests and t test were used in the study. Multiple linear regression analyses were used to probe factors associated with domains of sleep quality among undergraduate students. Several factors were considered in modeling the factors that affect the sleep quality. These factors included sex (male/female), grade (freshman/sophomore/junior), student leader (yes/no), nationality (minority/han), only child (yes/no), in love (yes/no), living costs (<500 CNY/500–800 CNY/800–1000 CNY/more than 1000 CNY), type of university (Comprehensive University/University of Foreign Languages/Medical University/Normal University/Technological University), and WeChat user (Yes/No). All statistics were performed using two-sided tests, and statistical significance was considered at p < 0.05. All data analyses were performed using a statistical software (SAS version 9.1; SAS Institute, Cary, NC, USA).

## Results

Of the 1979 participants, 1320 (66.70%) reported using WeChat. Significant differences between WeChat users and WeChat non-users were found in type of university (Comprehensive University: 6.6 vs. 33.4%; Medical University: 61.9 vs. 38.1%; Technological University: 66.2 vs. 33.9%; Normal University: 65.0 vs. 35.0%; University of Foreign Languages: 72.9 vs. 27.1%), grade (Freshman: 31.6 vs. 41.9%; Sophomore: 36.2 vs. 34.8%; Junior: 32.1 vs. 23.4%), student leaders (35.5 vs. 28.5%), courtship (32.7 vs. 27.2%) and monthly living expense (<800 Yuan: 60.2 vs. 39.8%; 800–1000 Yuan: 72.7 vs. 27.3%; more than 1000 Yuan: 76.8 vs. 23.2%). Results are shown in Table 1.

**Table 1:** The demographic characteristics of WeChat users and WeChat non users (n,  %)

Item	WeChat users	WeChat non users	p
Gender			<0.001*
Male	304 (23.0%)	231 (35.1%)	
Female	1016 (77.0%)	428 (65.0%)	
Age (years)	20.3 ± 1.2	20.2 ± 1.3	0.176
Grade			<0.001*
Freshman	417 (31.6%)	276 (41.9%)	
Sophomore	479 (36.2%)	229 (34.8%)	
Junior	424 (32.1%)	154 (23.4%)	
Whether the student leader	0.002		
Yes	469 (35.5%)	188 (28.5%)	
No	851 (64.5%)	471 (71.5%)	
Nationality			0.327
Minority	87 (6.6%)	36 (5.5%)	
Han	1233 (93.4%)	623 (94.5%)	
The only child	0.063		
Yes	705 (53.4%)	381 (57.8%)	
No	615 (46.6%)	278 (42.2%)	
In love			0.013
Yes	431 (32.7%)	179 (27.2%)	
No	889 (67.4%)	480 (72.8%)	
Living costs			<0.001*
<800	298 (60.2%)	90 (39.8%)	
800–1000	647 (72.7%)	428 (27.3%)	
More than 1000	375 (76.8%)	141 (23.2%)	
Type of university			0.012*
Comprehensive university	261 (66.6%)	131 (33.4%)	
Medical university	263 (61.9%)	162 (38.1%)	
Technological university	258 (66.2%)	132 (33.9%)	
Normal university	204 (65.0%)	110 (35.0%)	
University of foreign languages	334 (72.9%)	124 (27.1%)	

Chi square was used to compare differences in categorical variables, and t test was used to compare differences in continuous variables

* Statistically significant (p < 0.05)

Table 2 shows the multivariable analyses, which was used to predict the factors that influenced sleep quality. Compared with non-users, WeChat users had a lower score of subjective quality of sleep, sleep latency, use of sleeping medication, habitual sleep efficiency, daytime dysfunction, and global PSQI score. Compared with female, male had a lower score of subjective quality of sleep, sleep latency, habitual sleep efficiency, sleep disorders, daytime dysfunction and global PSQI score. However, females had a lower score of sleep duration than males. Age was not significantly associated with the domains of sleep quality. Compared with freshmen, sophomores had higher score of sleep duration. Compared with freshmen, sophomores had lower score of habitual sleep efficiency. Compared with freshmen, Junior had lower score of habitual sleep efficiency and global PSQI score. Respondents who with a living cost of ¥800–1000 had higher score of habitual sleep efficiency than those with less than ¥800. Compared with those was not the student leader, those were the student cadre had a lower score of global PSQI score. Compared with Han students, minority students had higher score of sleep duration but not the others. Compared with those were not the only child, those were the only child had a lower score of subjective quality of sleep, sleep latency, habitual sleep efficiency, sleep disorders, and global PSQI score. Compared with those were in love, those were not in love had higher score of habitual sleep efficiency. Compared with Comprehensive University, students from Medical University had a lower score of daytime dysfunction and habitual sleep efficiency. Compared with Comprehensive University, students from Technological University had higher score of sleep duration and had a lower score of daytime dysfunction and habitual sleep efficiency. Compared with Comprehensive University, students from Normal University had higher score of sleep duration and had a lower score of daytime dysfunction and habitual sleep efficiency. Compared with Comprehensive University, students from University of Foreign Languages had a lower score of daytime dysfunction, habitual sleep efficiency and global PSQI score.

**Table 2:** Multiple linear regression analysis for the factors affecting sleep quality in undergraduates in Chongqing, China

Parameter	Component 1		Component 2		Component 3		Component 4		Component 5		Component 6		Component 7		Global PSQI score	
	*B* (SE)	p	*B* (SE)	p	*B* (SE)	p	*B* (SE)	p	*B* (SE)	p	*B* (SE)	p	*B* (SE)	p	*B* (SE)	p
Male versus female	−0.12 (0.03)	*0.000*	−0.16 (0.05)	*0.001*	0.07 (0.04)	*0.046*	0.55 (0.08)	<*0.0001*	−0.12 (0.03)	**<** *0.001*	−0.02 (0.02)	0.470	−0.12 (0.04)	*0.008*	0.08 (0.14)	0.556
21–24 versus ≤20 years	0.04 (0.04)	0.204	0.10 (0.05)	0.076	−0.03 (0.04)	0.501	0.11 (0.08)	0.206	0.05 (0.03)	0.119	−0.00 (0.02)	0.926	0.02 (0.05)	0.653	0.29 (0.15)	0.052
25–28 versus ≤20 years	0.25 (0.35)	0.478	0.47 (0.51)	0.361	0.30 (0.38)	0.427	−0.83 (0.80)	0.298	0.45 (0.27)	0.100	0.20 (0.2)	0.387	0.23 (0.45)	0.609	1.06 (1.40)	0.448
Sophomore versus freshman	−0.04 (0.04)	0.235	−0.00 (0.05)	0.948	0.14 (0.04)	*0.000*	−0.31 (0.08)	*0.000*	−0.01 (0.03)	0.652	0.02 (0.02)	0.404	−0.07 (0.05)	0.120	−0.28 (0.14)	0.054
Junior versus freshman	−0.06 (0.05)	0.187	0.03 (0.07)	0.648	0.03 (0.05)	0.537	−0.35 (0.10)	*0.001*	−0.06 (0.04)	0.107	0.04 (0.03)	0.186	−0.10 (0.06)	0.082	−0.47 (0.18)	*0.011*
The student leader (yes vs. no)	−0.03 (0.03)	0.258	−0.09 (0.04)	0.046	−0.03 (0.03)	0.353	−0.02 (0.07)	0.783	−0.03 (0.02)	0.231	0.01 (0.02)	0.723	−0.06 (0.04)	0.105	−0.25 (0.12)	*0.036*
Minority versus han	−0.06 (0.06)	0.254	−0.01 (0.08)	0.939	0.23 (0.06)	*0.000*	0.06 (0.13)	0.670	−0.00 (0.04)	0.980	0.05 (0.04)	0.192	−0.16 (0.07)	0.031	0.11 (0.23)	0.633
The only child (yes vs. no)	−0.1 (0.03)	**<** *0.001*	−0.17 (0.04)	**<** *0.001*	0.03 (0.03)	0.300	−0.18 (0.07)	*0.006*	−0.09 (0.02)	<*0.0001*	0.03 (0.02)	0.094	−0.05 (0.04)	0.164	−0.53 (0.11)	<*0.0001*
In love (yes vs. no)	−0.04 (0.03)	0.221	−0.05 (0.04)	0.268	0.05 (0.03)	0.164	0.18 (0.07)	*0.009*	0.02 (0.02)	0.501	0.01 (0.02)	0.593	−0.08 (0.04)	0.049	0.09 (0.12)	0.448
Living costs (800–1000 vs. <800)	−0.01 (0.04)	0.740	0.04 (0.05)	0.505	−0.06 (0.04)	0.129	0.30 (0.09)	*0.000*	−0.02 (0.03)	0.413	−0.01 (0.02)	0.705	0.01 (0.05)	0.909	0.24 (0.15)	0.109
Living costs (more than 1000 vs. <800)	−0.00 (0.04)	0.937	0.12 (0.06)	0.045	0.01 (0.04)	0.760	0.00 (0.09)	0.993	0.04 (0.03)	0.231	0.04 (0.03)	0.160	−0.01 (0.05)	0.864	0.19 (0.16)	0.235
Medical university versus comprehensive university	−0.01 (0.05)	0.873	0.09 (0.07)	0.194	0.10 (0.05)	0.051	−0.24 (0.10)	*0.023*	−0.01 (0.04)	0.698	−0.03 (0.03)	0.266	−0.24 (0.06)	**<** *0.001*	−0.35 (0.18)	0.050
Technological university versus comprehensive university	−0.08 (0.04)	0.061	0.03 (0.06)	0.599	0.18 (0.05)	*0.000*	−0.20 (0.10)	*0.047*	0.06 (0.03)	0.113	0.05 (0.03)	0.106	−0.21 (0.06)	*0.000*	−0.18 (0.18)	0.315
Normal university versus comprehensive university	−0.03 (0.05)	0.535	0.03 (0.07)	0.679	0.11 (0.05)	*0.032*	−0.26 (0.11)	*0.017*	0.02 (0.04)	0.514	−0.00 (0.03)	0.98	−0.22 (0.06)	*0.000*	−0.35 (0.19)	0.071
University of foreign languages versus comprehensive university	−0.07 (0.05)	0.102	−0.19 (0.07)	0.003	−0.04 (0.05)	0.407	−0.44 (0.10)	**<** *0.000*	0.02 (0.04)	0.561	0.01 (0.03)	0.647	−0.16 (0.06)	*0.006*	−0.88 (0.18)	<*0.0001*
WeChat (use vs. not use)	−0.07 (0.03)	*0.038*	−0.11 (0.04)	*0.012*	−0.05 (0.03)	0.123	−0.75 (0.07)	**<** *0.000*	−0.03 (0.02)	0.244	−0.05 (0.02)	*0.005*	−0.18 (0.04)	**<** *0.001*	−1.23 (0.12)	<*0.0001*

Component 1: subjective quality of sleep, component 2: sleep latency, component 3: sleep duration, component 4: habitual sleep efficiency, component 5: sleep disorders, component 6: use of sleeping medication, component 7: daytime dysfunction

p values in italics denote statistically significant (p < 0.05)

## Discussion

This study found that WeChat users have better sleep quality in subjective quality of sleep, sleep latency, use of sleeping medication, daytime dysfunction, and global PSQI among undergraduates than non-WeChat users. Although higher social media use volume and frequency had significantly greater odds of having sleep disturbance among young adults (Levenson et al. 2016). Smartphone overuse may lead to depression and/or anxiety, which in turn leads to sleep problems (Demi̇rci̇ et al. 2015). Similarly, Huber et al. (2002) reported that electromagnetic field exposure (mobile phone usage) in the evening influences physiological factors such as sleep quality and the melatonin rhythm, probably by influencing the brain activity. Maybe WeChat users have a reasonable time arrangement about WeChat and time spent on WeChat at daytime is more than that at night especially before sleep. The use of WeChat has a limited effect on sleep. There are multiple environmental factors that affect their sleep–wake behavior, such as indulged in extracurricular activities (Millman 2005). WeChat users spent more time on WeChat than non-users therefore reduced the opportunity that indulged in extracurricular activities. Sleep quality is a mediator between technology use after sleep onset and depression and anxiety in college students (Adams and Kisler 2013). Sleep loss more generally has been associated with stress, depression and increased anxiety (Chorney et al. 2008). One reason is that WeChat could be used to relieve stressful situations and feelings of depression or anxiety. WeChat users may have better sleep quality because of depression relieving. Furthermore, non-users may also use other social media; these may affect the quality of their sleep. Microblogging is now considered an indispensable part of a college student’s life in China, and the excessive use of microblogs may bring about time-management problems (e.g., diminished quality and/or quantity of sleep time due to all-day use of microblogs) (Hou et al. 2014). Another possible explanation for these findings may be that participants use WeChat may have access to more resources and information, including the benefits of good sleep, more education may help to improve their sleep quality. Sleep education programs can ensure that sleep knowledge and the level of salience allocated to sleep health are increased so that any poor sleep habits may be more of a choice than a lack of awareness (De Sousa et al. 2007). College age students are particularly susceptible to engaging in unhealthy sleep hygiene practices, and thus could benefit from greater awareness of the importance of good sleep (Forquer et al. 2008). For example, many WeChat Subscriptions (a kind of WeChat public platform) promote some health knowledge. However, this result does not mean that excessive use of WeChat could result in better sleep quality. Because we didn’t incorporate every time spent on Wechat in our study, further research needs to be conducted to verify this result. It might help to increase the sleep quality and daytime functioning of these students. The study results suggest several other potential research avenues. For example, to acquire accurate the effect of WeChat on sleep quality among college students in general rather by institution, multi-site epidemiological cohort studies using validated screening tools and rigorous diagnostic criteria are needed.

This study also found that gender, grade, nationality, the student leader, the only child, living cost, type of university, in love were associated with domains of PSQI among undergraduates. Female college students may have more difficulty than male college students with sleep disturbances (Gaultney 2010). This is consistent with some other studies that found a significant association between gender and the poor sleep quality (Cheng et al. 2012). The third-year students did, however, have the lowest numerical mean global PSQI score and lowest prevalence of poor sleepers (Cates et al. 2015). Furthermore, short sleep appeared to be more common among minority race/ethnic groups, although the association was not significant (Whinnery et al. 2014). Student leader in University spent more time on work with high work demands and stress than common students. Barnes et al. (2012) found that time spent working is negatively associated with self-report sleep time. Children’s sleep disturbances were associated with their home environment that may be influenced by caregivers’ characteristics (Chen et al. 2014). The only child in the family usually got more attention than others about sleep health. Previous study found that economic stresses affect sleep–wake behavior of University students (Millman 2005). Although there might be significant variations from campus to campus, poor sleep quality and sleep deprivation were prevalent in university business students in Hong Kong, especially for those attending early morning lectures and living on-campus (Tsui and Wing 2009). In a Chinese study, 19% of the medical students were found to have poor sleep quality as assessed by PSQI, with differences seen between years of study but not between genders (Feng et al. 2005). In terms of self-reported short sleep, divorced and never married individuals were more likely than married respondents to report short sleep (Whinnery et al. 2014).

The results found that students from University of Foreign Languages were less likely to have poor sleep quality. This study further confirmed that female students in the sample were more likely to report poor sleep quality in Chinese undergraduates (Petrov et al. 2014). Besides, the majority of undergraduates in University of Foreign Languages in Chongqing are females which may influence the final result. Although there might be significant variations from campus to campus, college culture is characterized by social arrangements and behavior practices that negatively affect students’ sleep, including communal living, frequent parties and drinking, poor time management, and high levels of stress and work (Buboltz et al. 2002; Jensen 2003; Wechsler et al. 2000).

This study also has several limitations that need to be addressed. First, cross-sectional survey data reduced the ability of the study to make direct causal inferences, explore whether unmeasured factors can explain the observed relationships better, and determine the direction of causality. Second, this study did not conduct the psychometrics of the questionnaire. The future studies may include the psychometrics to assess sleep quality. Third, the sample is dominated by females. Fourth, we did have information about obesity that may confound the effect. Fifth, timing of use of WeChat is unknown and we did not evaluate the duration of WeChat in study. Future research is necessary to measure use of WeChat. Finally, the study was based on self-reports, which have their own limitations, as participants can be dishonest or not provide the correct responses. Notwithstanding the above limitations, this study also has certain strengths. This study is the first one to explore the effects of WeChat on the sleep quality of undergraduates in China. This exploratory study provides a workable framework for further exploration of the association between social media and sleeping problems, especially in China. This approach may help solve the increasingly serious sleeping problems of college students by providing a theoretical foundation for intervention.

## Conclusions

This study found that WeChat users may have better sleep quality in subjective quality of sleep, sleep latency, use of sleeping medication, daytime dysfunction, and global PSQI among undergraduates than non-WeChat users. This study is the first study to investigate the influence of WeChat on the sleep quality of undergraduates in China and that provides a workable framework for further exploration of the association between social media and sleeping problems particularly in China. This study suggests that social media (e.g., WeChat) can be used for health promotion to improve sleep or health related behaviors (such as exercise and eating habits). This research study holds significance for researchers and practitioners in health education and health promotion as it might provide suggestion for building prevention programs. To determine causal relationships, further longitudinal studies will be required to test for the association between WeChat users and sleep quality.
